# BNIP3L/NIX-mediated mitophagy: molecular mechanisms and implications for human disease

**DOI:** 10.1038/s41419-021-04469-y

**Published:** 2021-12-20

**Authors:** Yue Li, Wanqing Zheng, Yangyang Lu, Yanrong Zheng, Ling Pan, Xiaoli Wu, Yang Yuan, Zhe Shen, Shijia Ma, Xingxian Zhang, Jiaying Wu, Zhong Chen, Xiangnan Zhang

**Affiliations:** 1 Institute of Pharmacology & Toxicology, College of Pharmaceutical Sciences, Key Laboratory of Medical Neurobiology of The Ministry of Health of China, Zhejiang University, Hangzhou, China; 2 Key Laboratory of Neuropharmacology and Translational Medicine of Zhejiang Province, College of Pharmacology Science, Zhejiang Chinese Medical University, Hangzhou, China; 3 Department of Pharmacy, The First Affiliated Hospital, Zhejiang University School of Medicine, Hangzhou, China

**Keywords:** Cancer, Mitophagy

## Abstract

Mitophagy is a highly conserved cellular process that maintains the mitochondrial quantity by eliminating dysfunctional or superfluous mitochondria through autophagy machinery. The mitochondrial outer membrane protein BNIP3L/Nix serves as a mitophagy receptor by recognizing autophagosomes. BNIP3L is initially known to clear the mitochondria during the development of reticulocytes. Recent studies indicated it also engages in a variety of physiological and pathological processes. In this review, we provide an overview of how BNIP3L induces mitophagy and discuss the biological functions of BNIP3L and its regulation at the molecular level. We further discuss current evidence indicating the involvement of BNIP3L-mediated mitophagy in human disease, particularly in cancer and neurological disorders.

## Facts

- BNIP3L promotes mitophagy either via recruiting autophagosomes to target mitochondria or enhancing the formation of autophagosomes.
- BNIP3L-mediated mitophagy is regulated at the pre-transcriptional, posttranslational levels, and homodimerization of BNIP3L is required for its mitophagic activity.
- BNIP3L either participates in mitophagy independently or interplays with other autophagy/mitophagy pathways.
- BNIP3L involves in a variety of human diseases, including cancer, neurological, metabolic, and cardiovascular disorders.

## Introduction

Mitochondrial quality control plays a central role in cellular homeostasis. Besides mitochondrial biogenesis, mitochondrial elimination via the autophagosome-lysosome pathway, which is frequently termed mitophagy, plays a crucial role in maintaining the cellular steady state of mitochondrial functions [1, 2]. Mounting evidence indicated the significance of appropriate mitophagy, either in terms of extension and specificity, in cell development and human diseases. To meet the needs of mitochondrial turnover, mammalian cells evolved a variety of molecular machineries. In particular, a multitude of mitophagy receptors connects target mitochondria with autophagosomes for degradation by sensing distinct intracellular or environmental stress [3]. These mitophagy receptors can be regulated at different stages of expression, translation, and post-translation, thus are able to regulate mitochondrial elimination precisely.

BNIP3L/Nix is a mitochondrial protein from the outer membrane that belongs to the BH3-only protein from the BCL2 family. BNIP3L was initially recognized as a proapoptotic protein with milder efficacy in inducing apoptosis compared to other proteins in this family [4, 5]. These phenotypes raised concerns about the molecular function of BNIP3L besides inducing apoptosis [4, 6]. A milestone disclosure in this field was made by the discovery that mice with a *Bnip3l* gene deletion showed a dysregulated maturation of the reticulocytes, in which massive mitochondria accumulated due to an impaired mitophagic process [7, 8]. BNIP3L has been accepted as a mitophagy receptor that binds to Atg8 proteins [9]. Nevertheless, it is not yet fully understood how BNIP3L-mediated mitophagy is regulated at the molecular level. Hypoxia has been considered as a canonical stress factor that induces BNIP3L-mediated mitophagy since BNIP3L can be transcriptionally upregulated by the hypoxia-inducible factor-1alpha (HIF1A). However, BNIP3L upregulation was not substantial in some cell types despite hypoxic conditions [10–13]. Instead, other transcription factors and non-coding RNAs reportedly regulated the transcription of *Bnip3l* by sensing distinct environmental fluctuations. More intriguingly, emerging data indicated that posttranslational modification of BNIP3L, in particular phosphorylation and ubiquitination, finely regulates BNIP3L-mediated mitophagy [14–16].

The significance of BNIP3L-mediated mitophagy was initially emphasized in the development of reticulocytes [7]. Since then, accumulating evidence indicated the involvement of BNIP3L-mediated mitophagy in the development process of other cell types. The molecular mechanisms underlying BNIP3L-mediated mitophagy, however, have not yet been fully elucidated. BNIP3L might act as a mitophagy receptor by binding with LC3s; alternatively, it is possible that BNIP3L interplays with some other autophagy/mitophagy pathways, including BECN1, RHEB2, cardiolipin, and PRKN. Furthermore, it is now clear that problems in BNIP3L-mediated mitophagy are associated with a number of human diseases, including neurological disorders, metabolic diseases, and cancer. Unfortunately, due to a poor understanding of the association between BNIP3L-mediated mitophagy and different diseases, few studies addressed potential therapies. Here, we review BNIP3L-mediated mitophagy and discuss how this process is regulated at the molecular level. Furthermore, we update current perspectives on the role of BNIP3L in cell development and human diseases by integrating the findings from novel research studies.

## Mitochondria elimination by autophagy

**Fig. 1:**
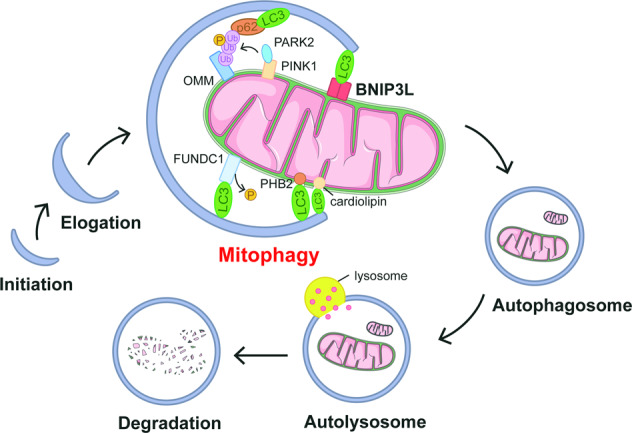
An overview of the mitophagy process. Autophagosomes are generated as phagophores and undergo elongation before ultimately fusing with lysosomes for degradation. Along with this process, mitochondria are recognized with the help from a variety of mitophagy-related proteins. The PINK1/PARK2 pathway senses the depolarization of mitochondria. The accumulation of PINK1 and PARK2 on the mitochondrial outer membrane amplifies the mitophagy signaling by generating phosphorylated ubiquitin, which links the autophagosomal machinery, e.g., P62 and LC3 to engulf the target mitochondria. Alternatively, a variety of mitophagy receptors, including BNIP3L, BNIP3, FUNDC1, PHB2, and cardiolipin, sense distinct extra-/intracellular signaling to recruit autophagosomes by directly binding with Atg8s, like LC3, via their LC3 interacting region (LIR) motif. These pathways ensure the superfluous and/or damaged mitochondria can be eliminated by lysosomes, and thus control the mitochondrial quality.

Autophagy maintains intracellular metabolic balance by degrading superfluous and damaged intracellular substances. The process of autophagy has been described elegantly [17]. Briefly, the biogenesis of autophagosomes is initiated by forming phagophores. Autophagosomal membranes are then further extended during maturation and fuse with lysosomes to form autolysosomes, in which the engulfed cargoes are degraded. Autophagy has been widely accepted as a selective, rather than random, process to eliminate cargoes [18]. Mitophagy is essential for mitochondrial quality control. Accumulating evidence indicates mitophagy dysregulation in a variety of human diseases. Mitophagy is restrictively controlled by several proteins, including PINK1/PARKIN, BNIP3L, and FUNDC1, etc. by sensing distinct extracellular signaling, e.g., mitochondrial depolarization, hypoxia, and cellular development (Fig. 1) [19]. Despite the fact that these mitophagy proteins ensure the basal mitophagy activity redundantly in intact cells, dysregulation of a given protein leads to specific disease-related phenotypes [20–23]. Given that most of the mitophagy-related proteins have been elegantly summarized elsewhere [24–26], the regulatory mechanisms of BNIP3L, as a mitophagy receptor, and its association with human disease have not been carefully reviewed.

## BNIP3L-mediated mitophagy

BNIP3L has been identified more than two decades ago [27, 28]. Sequencing analysis first recognized BNIP3L as a proapoptotic BCL2 family protein [28, 29] that could induce cell apoptosis or necrosis [30, 31]. Specifically, BNIP3L interacts with the proapoptotic Bcl2 family proteins BAX and BAK to increase the permeability of the mitochondrial outer membrane [32, 33]. This eventually leads to mitochondrial-dependent cell apoptosis. However, either endogenous or ectogenic BNIP3L expression has a poor influence on apoptotic cell death [34–36], compared to other BH3-only proapoptotic proteins [6]. BNIP3L has also been found in the endoplasmic reticulum (ER) [30], increasing its calcium storage through unknown mechanisms and promoting mitochondrial calcium uptake. The disruption of mitochondrial calcium homeostasis increases mitochondrial permeability and ultimately leads to cell demise [30, 37, 38].

Latterly the discovery of BNIP3L-promoted mitochondrial elimination by autophagy in developing reticulocytes opened a new horizon in BNIP3L research [7, 8], BNIP3L-induced mitophagy has since been identified in natural killer cells, neurons, retinal ganglion cells, renal cells, and several types of tumor cells [16, 39, 40]. In addition, BNIP3L might be involved in mitophagy in muscle and some cancer cells, even though direct evidence is still absent [41].

One central question is that how BNIP3L modulates mitophagy. BNIP3L disrupts the BCL2-BECN1 complex and releases BECN1, which promotes the formation of autophagosomes [42, 43]. BNIP3L also activates autophagy in epithelial cells from the respiratory tract [44]. Interestingly, BNIP3L-activated autophagy can be mimicked by a BH3 domain-like small molecule, which may serve as a promising drug [45, 46]. Furthermore, BNIP3L can inhibit MTORC1 to induce autophagy [47]. Interestingly, the gene plays a minimal role on the autophagy machinery of developing reticulocytes, ischemic brains, and hypoxic colon carcinoma [7, 16, 48]. Notwithstanding, BNIP3L plays a part in the regulation of autophagy activation, likely relying on the abundance of BECN1 or MTOR within distinct cell types.

**Fig. 2:**
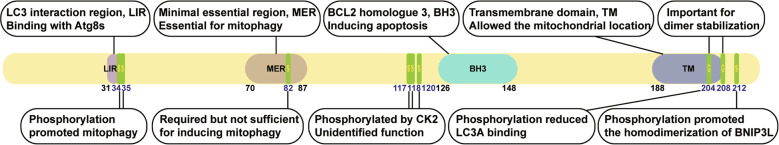
The regions and amino acid sites essential for BNIP3L biological functions. The LIR (LC3 interaction region) region binds with Atg8 proteins to induce mitophagy. The MER (Minimal essential region) is also essential for mitophagy. BH3 (BCL2 homolog 3) induces apoptosis. The TM (transmembrane domain) domain facilitates BNIP3L mitochondrial location. S34 and S35 phosphorylation promotes mitophagy. S82 phosphorylation is required but not sufficient to induce mitophagy. S117, S118, and S120 are phosphorylated by CK2, but their biological function remains unidentified. S204 and S108 are important for BNIP3L dimer stabilization. S204 and S212 phosphorylation promote BNIP3L homodimerization.

The most well-accepted mechanism underlying BNIP3L-mediated mitophagy is through an interaction with proteins from the Atg8 family, by which BNIP3L recruits autophagosomes to targeted mitochondria [9, 49]. Two BNIP3L domains are required for mitophagy, an LIR domain and a transmembrane TM domain (Fig. 2). The LIR domain is composed of four amino acid motifs that are critical for the interaction with LC3s. The BNIP3L genetic sequence contains two LIR motifs, of which the one located at the C-terminus [31–34] is essential for mitophagy. The deletion of the LIR motif in BNIP3L led to a failure to induce mitophagy [9]. However, it was later demonstrated that the deletion of a specific motif known as the minimal essential region (MER), rather than LIR, inhibited BNIP3L-mediated mitophagy [50]. These observations indicated that the MER, but not the LIR motif, is essential for BNIP3L-induced mitophagy. The TM domain located in the C-terminus of BNIP3L allows for its mitochondrial location, and the BNIP3L presence in other organelles leads to organelle-specific autophagy [51]. The TM domain does not participate in the interaction between Atg8 proteins and the LIR motif [49]. Recent studies indicated that BNIP3L may serve as a tag to label the targeted mitochondria for degradation [14, 16, 52], even though the current understanding of the processes regulating BNIP3L mitochondrial distribution remains limited.

Mitochondria are dynamic organelles undergoing continuous fission, fusion, and transportation. BNIP3L presence leads to the loss of mitochondrial transmembrane potential (ΔΨM) [30, 53–55], which was considered a marker of dysfunctional mitochondria that triggers mitophagy. Hence, BNIP3L seemingly induces ΔΨM collapse and thus initiates mitophagy. However, this assumption was challenged by the observation that BNIP3L does not necessarily cause ΔΨM loss when serving as a mitophagy receptor [7, 56]. Intriguingly, a recent study showed *Bnip3l* silencing can reduce ΔΨM during programmed mitophagy in cardiac progenitor cells (CPS) [57]. The causal relation among BNIP3L presence, ΔΨM loss and mitophagy should be further investigated. The PRKN/PARK2 can sense ΔΨM loss and ubiquitinate BNIP3L [58]. However, recent studies proposed BNIP3L does not functionally depend on PARK2 for manipulating mitophagy. In cells derived from patients suffering from Parkinson’s disease (PD), BNIP3L seemingly compensates for the loss-of-function mutation of PRKN/PARK2 and restores mitophagy [59]. Accordingly, BNIP3L (and not PRKN/PARK2) is involved in the programmed mitophagy of CPCs [57]. Furthermore, PARK2 deletion did not affect BNIP3L-induced mitophagy in ischemic neurons [16]. Finally, a recent study clearly demonstrated BNIP3L, rather than PARK2, is required for mitophagy clearance of axonal mitochondria [47]. These studies support distinct working mechanisms between BNIP3L and PARK2 and suggest ΔΨM loss may not be the most relevant trigger initiating BNIP3L-mediated mitophagy.

In summary, the role played by BNIP3L as a mitophagy receptor is now consensual, and extends beyond its function as a BH3-only proapoptotic protein. BNIP3L participates in mitophagy by promoting the formation of autophagosomes and, more directly, by recruiting them to target mitochondria. While the mechanistic details behind BNIP3L-mediated mitophagy need further elucidation, current evidence emphasizes BNIP3L is an independent mitophagy-inducing factor from the canonical PARK2.

## Regulations of BNIP3L-mediated mitophagy

Distinct mitophagy receptors may sense specific cellular stresses in order to initiate mitophagy. However, the specific environmental cues sensed by BNIP3L to regulate the mitophagic process remain unknown. Hypoxia is known intimately to signal BNIP3L-mediated mitophagy given HIF1A activates the transcription of *Bnip3l* [48, 60–62]. Paradoxically, this hypothesis originated from the studies in BNIP3, a homologous BNIP3L protein. Both *Bnip3* and *Bnip3l* were transcriptionally upregulated in CHO-K1 cells subjected to hypoxic conditions, but *Bnip3l* showed a considerably lower sensitivity to hypoxia [10], which was also observed in several tumor cell lines [11]. The assumption that hypoxia activates BNIP3L was further challenged by the observation that BNIP3L expression was neither related with pO2 in tumors [63] nor activated by ischemia in neurons or brain cells [16, 64]. In fact, hypoxia alone might not be sufficient to activate BNIP3L transcription [13, 35]. Some additional transcriptional factors such as TP53, FOXO3, PPARGC1A, E2F1, and FATE1 [34, 65–69], or other important growth factors [70] might be required for hypoxia-induced BNIP3L upregulation. Recent studies proposed that other cellular stresses or environment fluctuations are also involved in BNIP3L regulation. For example, BNIP3L transcription is activated by cytotoxic drugs [41, 71, 72], glucose level perturbations [73], and during the process of cell development [51, 56]. Mounting evidence indicated Bnip3l expression can be regulated by some micro RNAs, including miR-347, miR-30, miR-302/367, miR-133a, etc. [71, 74–76]. The regulation of BNIP3L by miRNAs was reviewed in previous work [77]. Recent data also proposed an epigenetic regulation of *Bnip3l* through DNA methylation [78, 79]. Therefore, BNIP3L expression can be regulated at both pre- and post-transcriptional levels and triggered by distinct cellular stresses including, but not limited to, hypoxia.

Current knowledge supports mitochondrial BNIP3L recruits autophagosomes needed for mitochondria degradation, which implies BNIP3L abundance is a decisive factor in mitophagy. However, BNIP3L is ubiquitously distributed on most mitochondria but only a portion of the BNIP3L-labeled mitochondria undergoes mitophagy. This paradigm is different from the PRKN/PARK2-induced mitophagy, in which PARK2 selectively accumulates on damaged mitochondria with low transmembrane potential to enable mitophagy. These observations raise the possibility that mechanisms other than location influence BNIP3L-mediated mitophagy.

Phosphorylation arguably remains the most carefully studied molecular mechanism behind BNIP3L-mediated mitophagy (Table 1). BNIP3L phosphorylation was initially found in a study identifying the CK2 substrate [80]. The BNIP3L serine residues S120, possibly S117 and S118 (by human sequence) as well, are phosphorylated by CK2, even though the functional significance of this phosphorylation remains unknown. Intriguingly, CK2 phosphorylates another mitophagy receptor FUNDC1 and prevented mitophagy under hypoxic conditions, suggesting an essential role of phosphorylation in receptor-mediated mitophagy [81]. Our previous study identified a serine 81 (S82 in human BNIP3L) phosphorylation is required for BNIP3L-induced mitophagy. The biological significance of S81 phosphorylation was confirmed in ischemic brain cells, in which a BNIP3L S81A mutant failed to eliminate damaged mitochondria and aggravated ischemic neuronal injury [16]. The S81 phosphorylation of BNIP3L was recapitulated in a cadmium-mediated mitophagy in HeLa cells [82]. Crucially, this residue is located in the MER motif region and might serve as an adapter essential for BNIP3L activity. The BNIP3L S81A mutant showed similar mitochondrial localization as the wild-type, but completely eliminated BNIP3L binding with LC3s and showed a limited enhancement of mitophagy induction [16]. This observation implied that unphosphorylated BNIP3L at S81 may act as a “silent” mitophagy receptor to avoid excessive mitophagic activity. The phosphorylation of the other two serine residues (S34 and S35), which are juxtaposed to the LIR motif of BNIP3L, showed a significantly higher affinity to LC3B [14]. In addition, phosphomimetic mutations of BNIP3L (S34, 35D or S34, 35E) reinforced its mitophagic activity. Interestingly, we found the BNIP3L S34, 35D mutation does not promote mitophagy in ischemic neuronal cells (unpublished data). These different pieces of evidence suggest BNIP3L phosphorylation occurring at distinct serine residues may play different functions in different cell types. A recent study by Novak et al. identified the phosphorylation at BNIP3L serine 212 residue promoted the homodimerization of BNIP3L to induce mitophagy [83], which is discussed below. Taken together, these data support a model where phosphorylated BNIP3L serves as a tag on mitochondria targeted for elimination among the BNIP3L-labeled mitochondrial pool. However, the specific phosphatases and kinases involved in controlling BNIP3L phosphorylation remain unknown, but these enzymes may constitute promising drug targets for treating human diseases associated with mitophagy dysfunction.

**Table 1:** Posttranslational modification (PTM) of BNIP3L and association with human disease.

Types	Sites	Biological functions	Related diseases	Ref.
**Phosphorylation**	S34, S35	S34/35 phosphorylation enhances autophagosome recruitment to mitochondria	Hela cells (Undetermined disease)	[14]
	S82	S82 phosphorylation is required for BNIP3L-mediated mitophagy	Cerebral ischemia	[16]
	S118, S120	Undetermined biological function	Undetermined related disease	[80]
	S212	S212 phosphorylation disrupts BNIP3L dimerization	HEK293 and HeLa cells (Undetermined disease)	[83]
**Ubiquitylation**	Unknown	Ubiquitination of BNIP3L by PARK2 recruits NBR1 to perform mitophagy.	Parkinson’s disease	[52]
	Unknown	Proteasomal degradation of ubiquitylated BNIP3L leads to mitophagy deficiency	Cerebral ischemia	[15]

Ubiquitylation is an alternative strategy for the selective elimination of BNIP3L-labeled mitochondria. BNIP3L can act as a PARK2 substrate, which is an E3 ubiquitin ligase. Ubiquitylated BNIP3L recruits the adapter protein NBR1 in the autophagosome to perform mitophagy [58]. This study thus provided an elegant illustration of how BNIP3L cooperates with PARK2 to induce mitophagy. We have also recently revealed an additional contribution of BNIP3L ubiquitylation to mitophagy regulation. Specifically, we identified a proteasomal degradation of ubiquitylated BNIP3L in ischemic brain cells that led to BNIP3L loss and mitophagy deficiency. The prevention of BNIP3L degradation by ubiquitin-proteasome inhibitors restored mitophagic activity and conferred neuroprotection [15]. We thus identified a novel mechanism through which BNIP3L-mediated mitophagy ceases under stressful conditions. We noted that the specific ubiquitylated BNIP3L lysine residues in these two scenarios remain unknown. This is also true for specific E3 ubiquitin ligases responsible for BNIP3L ubiquitylation besides PARK2. Such knowledge would help explore the distribution of E3 ligases within the mitochondria, particularly those associated with the regulation of mitophagy [84–86].

The transmembrane (TM) domain enables BNIP3L mitochondrial distribution, which is essential for its mitophagic activity. Importantly, the deletion of the BNIP3L TM domain does not affect its interaction with BCL2 but hampers the formation of homodimeric BNIP3L forms [27]. BNIP3L homodimers have first been described two decades ago [4, 27], but the biological function of dimeric BNIP3L remains undetermined. We recently showed dimeric BNIP3L is required for mitophagy [15]. Point mutations on the TM domain disrupt BNIP3L dimer formation without affecting its mitochondrial localization. A monomeric BNIP3L mutant failed to induce mitophagy in CCCP-treated HeLa cells and ischemic neuronal cells. Additionally, we found that the dimeric form of BNIP3L is more prone to degradation by the proteasomes in ischemic brains, suggesting an intrinsic mechanism through which neuronal cells tightly limit mitophagy. In accordance with this finding, recent work by Novak et al. supports the essential role of the BNIP3L dimer in mitophagy induction [83]. The same mutation in the TM domain (G204A) led to attenuated LC3A binding and mitophagy defects. Interestingly, the authors provided an elegant mechanistic illustration of the formation of the BNIP3L dimer. The phosphorylated serine 212 prevents BNIP3L dimer formation, indicating phosphorylation of distinct BNIP3L serine residues have opposite effects on mitophagic activity. Of note, the S212 residue is located in the intermembrane space of the mitochondria, suggesting mitochondria-derived signaling regulates mitophagy. These discoveries found an alternative BNIP3L control of mitophagy through the formation of homodimers. However, it is unclear how the BNIP3L dimer triggers mitophagy and the molecular mechanisms underlying BNIP3L dimerization.

**Fig. 3:**
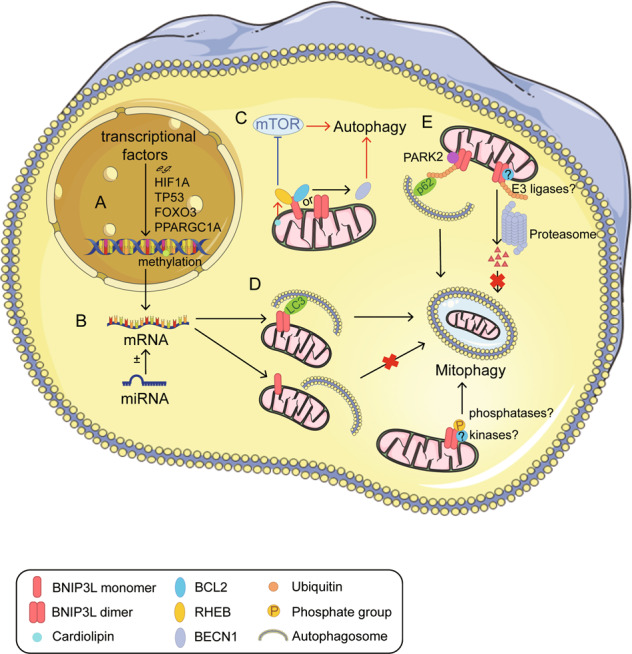
The regulations of BNIP3L and mitophagic activity. **A** Regulation of BNIP3L in the nucleus. BNIP3L is transcriptionally upregulated by several transcriptional factors, including hypoxia-inducible factor-1alpha (HIF1A), TP53, and FOXO3. In addition, DNA methylation ensures epigenetic regulation of BNIP3L. **B** Regulation of BNIP3L by micro RNAs (e.g., miR-347, miR-30, miR-302/367, and miR-133a). **C** The role played by BNIP3L in modulating autophagy. BNIP3L disrupts the BCL2-BECN1 complex; the released BECN1 subsequently promotes the formation of autophagosomes. Additionally, BNIP3L inhibits MTORC1 by prohibiting RHEB activity and subsequently induces autophagy. We note that the interaction between BNIP3L and RHEB can be enhanced by the accumulation of cardiolipin. **D** The role played by BNIP3L as a mitophagy receptor. The BNIP3L LIR motif interacts with LC3s to induce mitophagy. The BNIP3L monomer fails to induce mitophagy. BNIP3L phosphorylation at different sites regulates mitophagic activity but the associated kinases and phosphatases have not yet been identified. **E** BNIP3L dimer is required for mitophagy and is prone to degradation by the proteasome. PARK2 ubiquitinates BNIP3L and promotes mitophagy. Additional E3 ligases participating in BNIP3L degradation have not yet been identified.

BNIP3L-mediated mitophagy is finely controlled by several mechanisms beyond *Bnip3l* transcription (Fig. 3). Recent evidence indicated posttranslational modifications and homodimerization of BNIP3L are key participants in its mitophagic activity. Further research is necessary to understand how these BNIP3L modifications affect mitophagy as a response to environmental changes and their physiological/pathological significance.

## Crosstalk between BNIP3L and other autophagy/mitophagy pathways

Recent data indicated BNIP3L interplays with other autophagy-related pathways [42, 43, 52, 87]. Under hypoxia conditions, BNIP3L disrupts the BCL2–BECN1 complex by competitively binding with BCL2 via its BH3 domain, and the released BECN1 promotes the formation of autophagosomes [42, 43]. In a variety of cancer cell lines, BNIP3L regulation triggers autophagy and decreases cell death. Moreover, BNIP3L was shown to inhibit MTORC1 by preventing RHEB activity and subsequently induced autophagy. A recent study implied that dysfunctioned mitochondria are unable to sustain synaptic homeostasis and increase oxidative damage. The modulating effects of RHEB by BNIP3L in eliminating neuronal axonal mitochondria, showing potential benefits in counteracting Alzheimer’s [47].

Beyond interactions with autophagy-related pathways, BNIP3L also interplays with mitophagy pathways. Cardiolipin is essential for maintaining the structural organization and function of mitochondrial membranes [88], and the accumulation of cardiolipin is accompanied by the upregulation of BNIP3L. PLD6 knockdown prevents BNIP3L-induced MTOR-RPS6KB activation. The crosstalk between BNIP3L and cardiolipin provides a possibility to treat insulin desensitization. BNIP3 and BNIP3L possess common features with the BH3-only protein subgroup of the BCL2 family. Using a stringent, high-throughput yeast two-hybrid system, researchers found that BNIP3 interacts with BNIP3L [89]. However, existing research suggested that BNIP3 and BNIP3L mediate parallel death and autophagy pathways. In patients with breast cancer brain metastases and uveal melanoma, BNIP3 but not BNIP3L may serve as a putative biomarker [90, 91]. Also, previous studies suggested that BNIP3 may be a promising therapeutic target of myocardial infarction, insulin resistance, and osteoarthritis [92–94]. In cases of cell stress, ΔΨM loss can promptly occur and be sensed by the PRKN/PARK2, thus enabling the mitophagic process. BNIP3L was proposed as a PARK2 substrate that recruits adapter proteins in autophagosomes after being ubiquitinated. Briefly, the degradation of BNIP3L attenuates mitophagy and causes injury to DA neurons and PD [58]. Additionally, BNIP3L also interacts with other proteins such as DOK5, EWSR1, RINT1, and ADIPOQ [89, 95]. However, it is still unclear whether these interactions have biological significance.

## BNIP3L-mediated mitophagy in human diseases

Mitochondria play key roles in producing energy, buffering calcium, synthesizing steroids, and regulating programmed cell death [96]. The dysregulation of mitochondria is closely related to a variety of human diseases [97]. Neurons have extremely high requirements for energy. Mitochondria dysfunction is intimately associated with neurological disorders [15, 98–102]. Particularly, mitophagy defects may underlie the pathogenesis of Alzheimer’s disease, Parkinson’s disease, amyotrophic lateral sclerosis, and Huntington’s disease [103–107]. Moreover, it has been documented that mitophagy dysregulation plays discrepant role in tumorigenesis [108–110]. Therefore, it comes to a consensus that the disruption of mitophagy is highly involved in a variety of human diseases [97, 111, 112].

BNIP3L has been implicated in a variety of human diseases, including cancer, neurological, metabolic, and cardiovascular disorders. Mounting evidence supports the involvement of BNIP3L-mediated mitophagy in these disorders. Here, we focus on the role of autophagy/mitophagy activity of BNIP3L in human diseases by discussing recent studies in the field that shed light on the potential of targeting BNIP3L for therapeutics.

### Cancer

The contribution of BNIP3L to cancer cells remains controversial. BNIP3L is recognized as a tumor suppressor gene and a link between BNIP3L-related autophagy/mitophagy activity and cancer cell death has recently been investigated. BNIP3L reportedly caused autophagic melanoma cell death by recruiting TR3 to mitochondria [55]. The RNA oncomir gene mir-30d promotes tumor progression and impaired the expression of autophagy genes including *Bnip3l* [113]. Moreover, a study on glioma cells showed BNIP3L is required for the mitophagic cell death caused by the natural compound AT 101 [114]. Finally, another study showed endogenous BNIP3L degradation enabled the survival of Ewing sarcoma cells [68]. These observations showed that BNIP3L-mediated autophagy/mitophagy accelerated cancer cell death. Similarly, higher BNIP3L expression is associated with a better prognosis of acute myeloid leukemia (AML) and sensitized decitabine-induced cell demise without inducing apoptosis [115]. This process likely occurs via mitophagy regulation. In contrast, BNIP3L was considered as an oncogene that disrupts the interaction between BCL2 with BECN1. Accordingly, *Bnip3l* knockdown led to autophagy defects and increased cell mortality, which means BNIP3L-related autophagy might be related to survival in solid tumors in hypoxic environments [42, 43]. This idea was further supported by a transcriptome analysis indicating BNIP3L upregulation is closely linked with TNF-α resistance in breast cancer cells [116]. Additionally, BNIP3L was found to introduce XIAP into mitochondria through a yet-unidentified mechanism to degrade SMAC. This process can delay mitochondrial-dependent apoptosis in MCF-7 cells [32]. A growing amount of evidence showed that mitochondrial BNIP3L promotes the survival of glioblastoma cells and pancreatic cancer cells. BNIP3L-induced mitophagy alternates either glucose metabolism or mitochondrial OXPHOS status to adapt to the oncogenic environment [117, 118]. In AML cells, *Bnip3l* silencing leading to increased sensitivity to mitochondria- but not DNA-targeting drugs, also implies a potential link between BNIP3L-mediated mitophagy and cancer cell survival [119].

It must be emphasized that mitophagy reportedly plays a dual role in cancer biology [26, 120]. The level of BNIP3L is high in organoids derived from murine pancreatic intraepithelial neoplasia, thereby delaying progression of pancreatic ductal adenocarcinoma (PDAC) and suggests BNIP3L-mediated mitophagy promotes tumorigenesis [118]. However, a greater amount of evidence from different cancer cell lines and animal models showed BNIP3L-mediated mitophagy alters with the stage of tumor progression.

We also noted that BNIP3L-mediated mitophagy might play a role in anti-cancer treatment by regulating the formation of immunological memory. There is evidence that BNIP3L-mediated mitophagy is able to remove dysfunctional mitochondria and ROS accumulated in natural killer cells when proliferating. This is thought to promote cell survival during the transition to memory cells [39]. In CD8 + T cells, BNIP3L-mediated mitophagy deficiency causes superoxide accumulation in mitochondria and reduction of ATP synthesis, which inhibits the formation of effector memory and recall response [121].

**Fig. 4:**
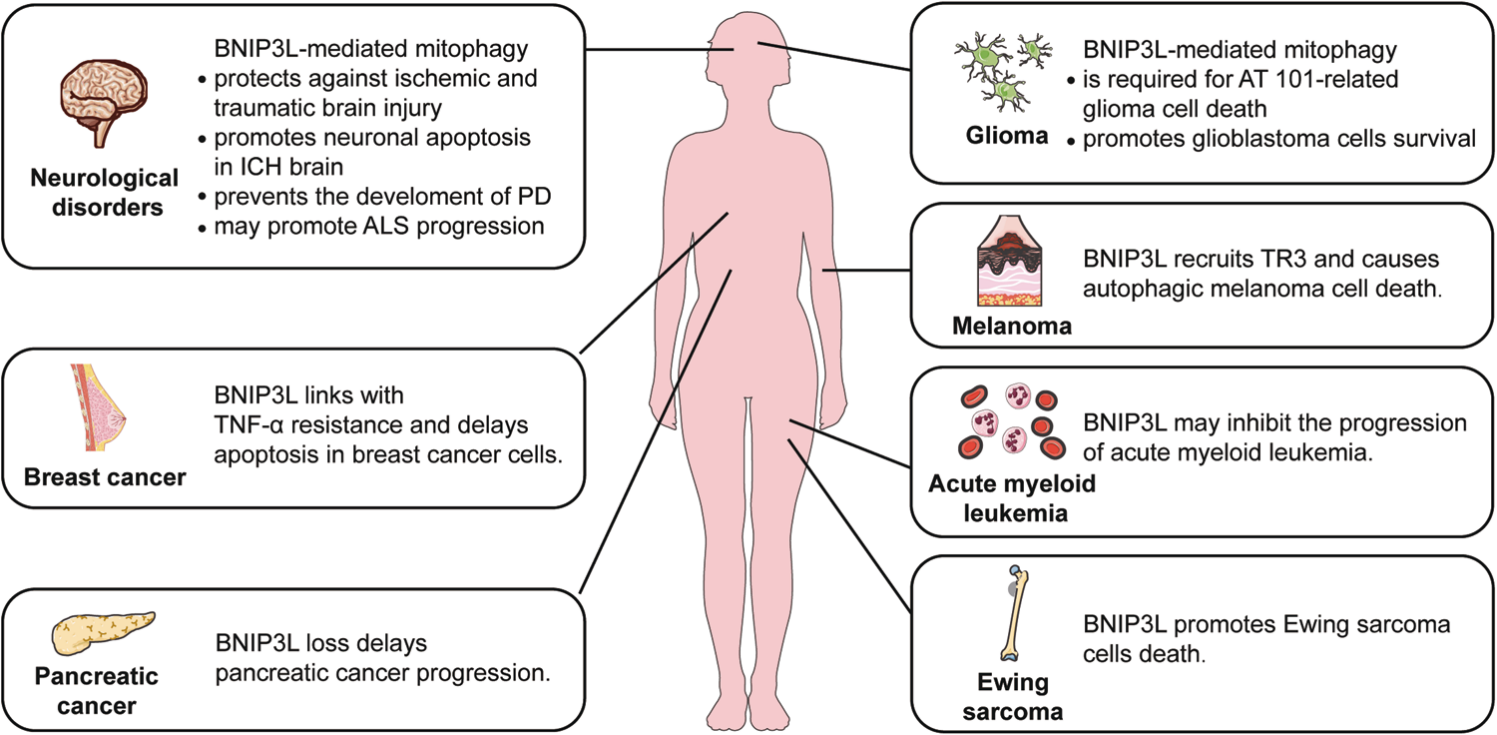
The implications of BNIP3L in human diseases.

Taken together, BNIP3L-mediated autophagy/mitophagy plays a complex role in the fate of cancer cells (Table 2 and Fig. 4). This complexity can be attributed to the heterogeneity of different types of cancer, the exposure to anti-cancer drugs, the distinct stages and dynamic niches of cancer progression, and the formation of immunological memory. Hence, it is still a pending question whether disturbing BNIP3L-mediated mitophagy represents a safe and efficient strategy in cancer therapy.

**Table 2:** The involvement of BNIP3L in human disease.

Diseases		Evidence	Ref.
**Cancer**	Melanoma	BNIP3L causes autophagic melanoma cell death by recruiting TR3 to mitochondria	[55]
	Glioma	BNIP3L participates in the mitophagic glioma cell death caused by AT 101	[114]
	Ewing sarcoma	Degradation of endogenous BNIP3L is required for the survival of Ewing sarcoma cells	[68]
	Acute myeloid leukemia	BNIP3L expression as a useful prognostic marker of acute myeloid leukemia	[115]
	Solid tumor (cell lines)	Autophagy via BNIP3L promotes tumors	[42, 43]
	Breast cancer	Upregulation of BNIP3L linked with TNF-α resistance	[32, 116]
		Upregulation of BNIP3L delays mitophagic apoptosis	
	Glioblastoma	BNIP3L-induced mitophagy reduces oxidative damage and promotes glioblastoma cell survival	[117]
	Pancreatic cancer	Deletion of *Bnip3l* delays the progression of pancreatic cancer	[118]
**Neurodegeneration**	Parkinson’s disease	BNIP3L compensates for mitophagic defects in PD patient-derived cells	[59, 122]
	Amyotrophic lateral sclerosis	Upregulation of BNIP3L may promote ALS progression via a non-apoptotic function	[127]
**Acute brain injury**	Cerebral ischemia	BNIP3L protects against cerebral ischemia via mitophagy. The inhibition of BNIP3L degradation by carfilzomib attenuates cerebral ischemia.	[15, 16]
	Traumatic brain injury	*Bnip3l* overexpression enhances autophagic flux and attenuates traumatic brain injury	[60]
	Intracerebral hemorrhage	BNIP3L is upregulated in intracerebral hemorrhage rats. The increased interaction between BNIP3L and p75 promotes neuronal apoptosis	[137, 138]

### Neurodegeneration

Mitophagy has been recognized as essential for neuronal survival during neurodegeneration. The discovery that Parkinson’s disease (PD)-related genes *Pink1* and *Park2* participate in mitophagy highlighted how this process maintains the steady state of striatum dopaminergic neurons. However, *Park2* deletion failed to recapitulate PD phenotypes in mice, implying other compensatory mechanisms exist to ensure mitophagy [122, 123]. While it is still debatable whether PARK2 is involved in BNIP3L-mediated mitophagy, recent evidence implied BNIP3L may compensate for mitophagy in PD. Accordingly, an MPTP-induced parkinsonism model BNIP3L was downregulated, but this could be reversed through neuroprotectant rescuing dopamine neuron death [124]. In a murine model carrying a GBA mutant, which is a genetic risk factor for PD, the BNIP3L dimer was reduced and this was accompanied by mitophagy dysfunction. Of note, the GBA mutation did not lead to PARK2 and ubiquitin alternations, implying less involvement of PARK2 in mitophagy defects [125]. In a murine model carrying a GBA mutant, which is a genetic risk factor for PD, the BNIP3L dimer was reduced and this was accompanied by mitophagy dysfunction. Of note, the GBA mutation did not lead to PARK2 and ubiquitin alternations, implying less involvement of PARK2 in mitophagy defects [59]. Even though these data suggested BNIP3L acts as an alternative molecular pathway restoring mitophagy in PD, it is still unclear whether BNIP3L is able to rescue dopaminergic neuronal death and PD symptoms, especially in the sporadic cases of PD.

BNIP3L upregulation was found in the cerebrospinal fluid of patients suffering from amyotrophic lateral sclerosis (ALS), a degenerative disorder of the motor neurons [126]. Likewise, BNIP3L upregulation was documented in the astrocytes of SOD1 mutant mice, a widely used model for ALS [127]. It is unraveled that whether upregulated BNIP3L can increase mitophagy activity, although some papers denoted that mitophagy defects might underlie ALS pathology [128, 129]. Besides PD and ALS, other studies indicated the potential involvement of BNIP3L-mediated autophagy/mitophagy in other neurodegenerative conditions, but the evidence is not conclusive [47, 130, 131]. Finally, there is no evidence indicating an association between *Bnip3l* mutation and neurodegeneration, although an emerging preliminary study implied its involvement in schizophrenia [132]. As compensation for mitophagy defects, it is likely that enhancing BNIP3L-mediated mitophagy offers a potential therapeutic strategy to treat neurodegenerative disorders.

### Acute brain injury

Current evidence indicates BNIP3L is involved in acute brain injury disorder including cerebral ischemia, intracerebral hemorrhage (ICH), and traumatic brain injury, while most evidence indicates a proapoptotic role of BNIP3L. However, recent studies shed light on BNIP3L-mediated mitophagy in these diseases. The first report indicating BNIP3L upregulation and neuronal apoptosis in ischemic brain cells dates from 2005, before the gens was recognized as a mitophagy receptor [133]. Enhanced binding of BNIP3L with LC3 was observed in a delayed manner (72 h) after acute brain ischemia, suggesting excessive mitophagy may be correlated with delayed neuronal death [134]. Nevertheless, the contribution of BNIP3L-mediated mitophagy to brain ischemia was not determined by silencing or deleting the *Bnip3l* gene in animal cells. Our previous work revealed a neuroprotective role of mitophagy in acute brain ischemia in *Bnip3l* knockout mice [135, 136]. We demonstrated BNIP3L is required for ischemia-reperfusion-induced neuronal mitophagy and BNIP3L-mediated mitophagy offers protection against ischemic stroke by reinforcing clearance of damaged mitochondria [16]. In line with these findings, BNIP3L was found to be degraded by the proteasomes in permanent ischemic mice brains, which further leads to mitophagy defects. Blocking BNIP3L loss with the proteasomal inhibitor drug carfilzomib restored mitophagy and attenuated ischemic stroke [15]. These data emphasized the benefits of BNIP3L-mediated mitophagy in acute brain ischemia. Similarly, in a model evaluating traumatic brain injury in rats, neuronal BNIP3L reduction was found within 24 h after trauma. The overexpression of *Bnip3l* enhanced autophagic flux and attenuated traumatic brain injury, but it is still unclear whether mitophagy was also activated [60].

In contrast to studies on ischemic and traumatic brain injuries, there is a paucity of data indicating the involvement of BNIP3L-mediated mitophagy in intracerebral hemorrhage. It was shown that BNIP3L undergoes transient upregulation after brain hemorrhage in rats. However, the BNIP3L binding with BCL2 remained intact, suggesting minimal impacts on autophagy induction [137]. Alternatively, the bond between BNIP3L and p75 might stabilize BNIP3L on the outer mitochondrial membrane and promote neuronal apoptosis in the ICH brain [138]. However, these findings were argued since only a few neuronal cells showed features of apoptotic or autophagic death after ICH [139].

Overall, there is no strong evidence demonstrating a fundamental role of BNIP3L in any specific disorders, and there are no existing clinic approaches targeting BNIP3L. However, current evidence supports BNIP3L is involved in a variety of human diseases, from cancer to neurological disorders. Besides, BNIP3L-mediated mitophagy also involves in retinal ganglion cell development, intestinal inflammation, adult cardiac progenitor cells differentiation, and myocyte insulin resistance [57, 140–142]. Interestingly, an emerging study revealed BNIP3L mutations in human schizophrenia [132]. These studies highlighted the potential value of BNIP3L as a promising biomarker and drug target for specific human diseases.

## Perspectives

It is increasingly clear that BNIP3L induces mitophagy by either activating autophagy or serving as a mitophagy receptor. Several studies showed BNIP3L is involved in diseases such as cancer, neurodegenerative disorders, and acute brain injury. BNIP3L plays a dual role in different kinds of cancer progression. In solid tumors (cell lines), breast cancer, glioblastoma, and pancreatic cancer, BNIP3L exacerbate cancer progression. However, it has an inverse role in the case of acute myeloid leukemia, melanoma, glioma, and Ewing sarcoma [32, 42, 43, 55, 68, 114–119]. These contrasting roles might be related to the unique metabolic environments of different tumors, but remained unclear. Additionally, it was reported that mitophagy also plays dual roles at different stages of cancer progression, despite a lack of studies addressing this specific question. In the case of neurodegenerative diseases such as Parkinson’s and ALS, BNIP3L accelerated disease progression [59, 122, 127]. However, due to the complexity of neurodegeneration and current technical limitations, it is not yet possible to thoroughly understand the underlying mechanisms. In the case of acute brain injury, BNIP3L protects the brain from damage. We found this is primarily related to BNIP3L-mediated mitophagy and independent of the PRKN/PARK2 [16]. We also showed BNIP3L dimer mediates mitophagy activity [15]. However, there are several important and outstanding questions in this case, including (but not limited to): (1) What are the key extra- and intracellular stimuli that trigger BNIP3L-mediated mitophagy? (2) How does BNIP3L regulate the extension and selectivity of mitophagy by sensing these stimuli? (It is possible BNIP3L responses to stimuli during phosphorylation and other posttranslational modifications). Nevertheless, the key amino acid residues and enzymes responsible for phosphorylation remain elusive. The identification of these enzymes might bring forward promising drug targets for BNIP3L-related human diseases, particularly neurological disorders. Unlike the case of PRKN/PARK2 and Parkinson’s disease, there is a paucity of data supporting the association between BNIP3L mutations and specific diseases. Given the known involvement of BNIP3L-mediated mitophagy in development and diseases, it is essential to identify key BNIP3L mutations to understand the biological function of this gene and provide the rationale for discovering novel therapeutic strategies.

## Data Availability

The data used to support the findings of this study are available from the corresponding author upon request.
